# AIT in pediatric allergology: Opportunities and difficulties on the home stretch of the Therapy Allergen Ordinance 

**DOI:** 10.5414/ALX02443E

**Published:** 2023-12-12

**Authors:** Christian Vogelberg, Michael Gerstlauer

**Affiliations:** 1 Department of Pediatrics, Faculty of Medicine and University Hospital Carl Gustav Carus, Technische Universität Dresden, Dresden, and; 2 Pediatric and Adolescent Medicine, University Hospital Augsburg, Augsburg, Germany

**Keywords:** Therapy Allergen Ordinance, allergen immunotherapy, asthma, allergic rhinitis, children, supply gap, dose-finding studies, placebo-controlled study, efficacy, sublingual, subcutaneous

## Abstract

Allergen immunotherapy (AIT) is the only causal therapy for allergic diseases and therefore particularly important. Allergen preparations have been classified as medicinal products since 1989 (Directive 89/342/EEC) and were taken over into Directive 2001/83/EC in 2001. In addition, in 2008 the Therapy Allergen Ordinance (TAO) came into force to stricter regulate the exception for named patient products (NPP) by exclusion of common therapy allergens from the exception to be marketed as NPP. The TAO regulates the requirements for testing safety and efficacy for these common therapy allergens. Due to the long transitional provisions, the last deadlines for solving clinical shortcomings will end in 2026. The advantage of this regulation is that the market for common allergens has been cleared of products without proof of efficacy, and new preparations with an optimal dose range are developed through dose-finding studies. The demand for long-term pediatric studies has been outlined by the standard Pediatric Investigation Plan (PIP) on allergen products from the Pediatric Committee of the EMA (PDCO). This is particularly problematic, as it is foreseeable that recruitment of patients will be limited and ethical problems arise from the prolonged use of placebo. Furthermore, many newly approved preparations will not be used in pediatrics for the foreseeable future, as no marketing authorization has yet been granted for this age group. This will result in a serious supply gap for children.

## Introduction 

Allergies and atopic diseases have existed for years with a high prevalence not only in adults, but also especially in children. For example, the 12-month prevalence of allergic sensitization in children and adolescents in Germany is ~ 37%, the lifetime prevalence of asthma is 6%, of allergic rhinitis 11%, and of atopic dermatitis ~ 13% [1]. These diseases are thus among the most common chronic diseases in childhood and adolescence. 

Treatment focuses on the pathophysiology of inflammation and consists mainly of allergen avoidance and symptomatic therapy. Allergen immunotherapy (AIT) is the only form of therapy that follows a causal treatment approach, as its goal is not primarily symptom control, but immunomodulation [2]. Due to the development of tolerance, AIT also has significant positive effects on clinical symptoms and medication consumption [3, 4] and alleviates symptoms more effectively than symptomatic therapy alone [5]. Meta-analyses show this for the treatment of allergic rhinitis [6, 7] as well as for bronchial asthma [8, 9]. 

In addition, there is now increasing evidence that AIT has preventive effects on the course of the disease, especially in patients with allergic rhinitis. After AIT, they develop bronchial asthma significantly less frequently, and also significantly less new allergic sensitization occurs [10, 11, 12]. Children in particular seem to benefit from AIT and respond well to it. In the few clinical trials in which identical preparations were investigated in children/adolescents and adults, comparable efficacy and safety were shown in younger subjects [13, 14]. This was also confirmed in European Real World Evidence (RWE) studies [3, 15]. 

Despite the successful history of AIT, which is now more than a century old, the improved understanding of its mode of action and the growing evidence of its potential efficacy, there are comparatively few studies on its use, especially in children. The composition and content of the various AIT preparations vary significantly worldwide, as do the rules regarding the approval of the preparations. Within the EU the requirements within a marketing authorization (MA) process are harmonized. In Germany, the Therapy Allergen Ordinance (TAO) was initiated due to exploited exemption rules for named patient products (NPP) in the German Medicinal Products Act for common allergens. It was and is associated with considerable consequences for pharmaceutical companies and their products. Further regulatory efforts also exist at the European level to achieve harmonization [16]. 

## Core paper 

Since 1989 all AIT products have been defined as medicinal products and subject to authorization in Germany with exemption of individual prescriptions (NPPs). Many of the predominantly used AIT products containing common allergens in Germany were authorized during the 1970s, 1980s, and 1990s. With the 14^th^ amendment to the German Medicinal Product Act due to the change in the definition of a “finished medicinal product” also individual prescriptions would have required a marketing authorization. To save true individual products especially for the treatment of allergies caused by rare allergens, the Section 21 (2) no. 1g was included to exempt AIT products on individual prescription from marketing authorization [17]. On November 11, 2008, the TAO came into force. However, this does not affect all therapy allergens that can be included in an AIT product but only therapy products with common allergens (grass, birch, alder, and hazel pollen, house dust mites, and insect venoms). AIT products made individually for a patient with allergens from other sources are not subject to this marketing authorization requirement [18]. After the transitional period, only authorized AIT products can be used for AIT against the above-mentioned common allergen sources. By May 14, 2009, manufacturers had to indicate for their NPPs which contained one or more of the above-named common allergen sources whether they intended to apply for MA, or whether no MA was planned. Five months later, the official batch release testing of bulk material of these NPPs began. In 2010, 123 applications for marketing authorization had been received by the Paul-Ehrlich-Institut (PEI) [18]. As of July 3, 2023, 43 procedures out of 123 were still running [19]. The last product-specific deadlines under the provision of TAO will end in 2026. If the necessary data are not submitted by the manufacturer within the granted product-specific period, the marketability of the respective NPP will end. Regardless of the MA status, all products containing common allergens listed in the TAO are subject to batch testing at the PEI [20]. 

What are the concrete consequences for the therapeutic allergen prescription, what are the chances and disadvantages of the TAO? A positive effect of the TAO was the associated reduction of the previously high supply of partly nonsensical combinations and ineffective low allergen dosages of the preparations. From originally 6,654 notifications, 123 were submitted for MA of which 43 are still running and 2 were authorized after development as new products. In particular, the study-based identification of the optimal therapeutic dose is an urgently needed procedure that is also standard for other medicines. However, there is a lack of reliable efficacy and safety data from placebo-controlled studies for most of the products that are still marketable so that this gap urgently needs to be closed. This applies also for children and adolescents. The Pediatric Investigation Plan (PIP) is a legal requirement which is laid down in regulation EC 1901/2006 of the European Parliament on medicinal products for pediatric use [21]. A standard PIP for allergen extracts for specific immunotherapy has been developed by the Pediatric Committee (PDCO) of the EMA [22]. 

The changes and requirements resulting from the TAO result in multiple problems for practical implementation in pediatric allergology, some of which are still the subject of intensive discussion. One particular problem is the requirement for pediatric studies. According to the PDCO, a double-blind, placebo-controlled, randomized (DBPCR) study over 3 years with 2 years of follow-up should be conducted for one so-called “selected product” of each manufacturer. After successful performance of this study, all other PIPs of this manufacturer can be modified and switched to short-term studies [22]. In practice, the problem arises that the recruitment of the necessary number of patients fails due to the lack of willingness of the families to participate. In particular, the aspect of receiving regular placebo doses over 3 years is perceived as unacceptable by many relatives. In addition, there is the ethical problem that AIT is widely recognized as a causal therapy, although data supporting this aspect are rare. However, both controlled studies and real-world evidence (RWE) data demonstrate a disease-modifying and also preventive effects on the further expansion of allergic sensitization and the development of bronchial asthma in existing allergic rhinitis [10, 11, 12]. Against this background, a 3-year therapy with a placebo plus a 2-year follow-up justifies further discussions about possible consequences through to a delay of an effective therapy by 5 years. This problem has already been identified and options for a solution have been discussed, but so far without any trend-setting results:

- Studies on one allergen source with preparations from several manufacturers in different therapy arms and a common placebo arm, have been proposed to minimize the number of children receiving placebo. Advantage: reduction of the placebo group Disadvantage: placebo group does not receive effective therapy
- In polyallergic children, AIT with one allergen A without therapy against a second allergen B could be compared with AIT against allergen B without treatment against allergen A. Advantage: no placebo administration Disadvantage: no simultaneous therapy for both relevant allergens, no efficacy data superior to placebo can be obtained.
- For long-term studies, a staggered treatment would be appropriate. Group 1 could receive active treatment for 3 years and then followed up for 2 years. Group 2 first receives placebo for 1 year, then active treatment for 3 years and is followed up for another year. Advantage: no group remains untreated, shortened placebo administration. Disadvantage: no reliable efficacy data on a carry-over effect, lack of long-term safety data comparing active treatment/placebo.
- After MA for the indication “treatment of allergic symptoms” in allergic rhinitis, with proof of safety and efficacy in short-term studies, the long-term effects can be recorded and evaluated via RWE data. Advantage: no placebo administration, large number of cases, no artificial framework. Disadvantage: no standardized framework, no direct recording of target parameters, no efficacy data superior to placebo as analyzed in DBPCR studies can be obtained.

Nevertheless, one approach to obtain supportive pediatric data could be the inclusion of RWE data, which are obtained, for example, from health insurance registers. The advantage of these data lies in the large number of patients that can be included in the analyses, their representativeness due to the lack of artificial selection of inclusion criteria, and the larger time periods to which the analyses can be extended, if a reasonable quality of data can be assured. Based on literature search for retrospective cohort assessments of prescription databases in Europe, an overview on the methodology, long-term effectiveness outcomes, and adherence to AIT has recently been provided [12]: AIT was more effective in reducing the progression of allergic rhinitis compared to a non-AIT control group receiving only symptomatic treatment for allergic rhinitis for up to 6 years. AIT also influenced the development and progression of asthma in most endpoints and most preparations in those patients who were treated compared to those who received only anti-asthmatic medication. The analysis of long-term effectiveness outcomes of the RWE studies based on prescription databases confirmed the long-term efficacy of AIT demonstrated in RCTs. The disadvantage that can occur due to a certain diagnostic imprecision in patient selection is compensated for by the large number of patients. This form of study definitely does not replace the gold standard of the DBPCR study but could deliver valuable supportive data from the pediatric population. 

Without data from pediatric studies, a central problem arises for the care of allergic children and adolescents, since treatment of pediatric patients would result in an off-label use. After completed studies in adults, pediatric MA is initially lacking for both NPP under the provision of TAO and completely new developed preparations which were not on the market as NPPs before [23]. The original (old) product, under the provision of TAO, will no longer be marketed by the companies, resulting in an increasing supply gap as documented in Table 1. Treatment with the preparation authorized for adults only would represent an off-label therapy for children and adolescents, the reimbursement of which would not be guaranteed and would result in an increased liability risk for the prescriber. 

According to the framework guidelines of the National Association of Statutory Health Insurance Physicians together with the National Association of Statutory Health Insurance Funds, since 2021 only authorized therapy allergens are to be used for new initiations of AIT against common allergen sources listed in the TAO. This national requirement has now been implemented regionally by the Associations of Statutory Health Insurance Physicians as a prescription recommendation [24, 25]. Marketable therapeutic allergens can continue to be used for the continuation of ongoing treatments. 

Exceptions to these restrictions exist only for therapies with rare allergens. “Rare allergens” are allergens that do not fall under the TAO. They can still be prescribed as individual prescriptions without MA. Examples are tree pollens that do not belong to the birch family (e.g., ash), summer herb pollens, mold spores, storage mites, or animal dander. Only a few studies exist for these allergen sources. Accordingly, the evidence for efficacy is lower and the recommendations for therapy in the AIT guideline more cautious [2]. 

Last product-specific deadlines under the provision of TAO will end in 2026. Clinical studies within the development program of TAO need to be finalized and submitted before expiration of deadlines. 

## Conclusion 

The TAO is an opportunity to clear the market of AIT preparations that either make no therapeutic sense or for which no efficacy and safety data are available. With the reduction to products with new data on efficacy and safety, a fundamentally effective and causal form of therapy can henceforth be used and unnecessary costs can be avoided. 

This demand on the manufacturers is high but necessary against the background of the regulation described above. But not only the companies have to submit the required studies; the authorities also have to move forward quickly with the assessment within the legal framework. And last but not least, study centers must be identified that can master the challenges of patient identification, approach, and recruitment. Whether the required number of pediatric study patients can be recruited at all remains to be discussed. 

For pediatric care, ethical and structural problems are foreseeable with regard to the studies required in their current form, which makes a revision of the current PIP necessary. 

## Funding 

No funding. 

## Conflict of interest 

Christian Vogelberg claims to have received research support/study funds for his hospital from the following companies in the last three years: Allergopharma, Boehringer Ingelheim, DBV Technology, Novartis Pharma. Lecture and consultancy fees have been received from Ärzteverband Deutscher Allergologen, Aimmune Therapeutics, ALK-Abelló, Allergopharma, Allergy Therapeutics, AstraZeneca, Bencard Allergie, Gesellschaft Pädiatrische Allergologie und Umweltmedizin, LETI Pharma, Novartis, Orion Pharma, Sanofi Aventis, Stallergenes-Greer in the last three years. There are memberships in the following professional societies: GPAU, GPP, EAACI, ERS, DGKJ, APPA. 

Michael Gerstlauer claims to have received research support/study funds for his hospital from the following companies in the last three years: Allergopharma, Bencard Allergie, Stallergenes-Greer, Boehringer Ingelheim, Infectopharm. Lecture and consultancy fees have been received from ALK-Abelló, Allergopharma, Bencard Allergie, Leti, Novartis, Sanofi, Stallergenes-Greer in the last three years. There are memberships in the following professional societies: GPAU, GPP, EAACI, ERS, DGKJ, AGPAS. 

**Table 1.** Allergen immunotherapy products authorized for children and adolescents in Germany containing common inhalant allergen sources.

	Children (5 to 11 years)		Teenagers (12 to 17 years)	
	SCIT	SLIT	SCIT	SLIT
Grass pollen	**ALK depot SQ Allergovit Purethal TA top**	**Grazax Oralair**	**ALK depot SQ Allergovit Purethal TA top**	**Grazax Oralair**
Tree pollen (birch/alder/hazel)	**ALK depot SQ Allergovit Purethal TA top**	*	**ALK depot SQ Allergovit Purethal TA top**	*
House dust mites	**ALK-depot SQ Depigoid Novo-Helisen**	*	**ALK-depot SQ Depigoid Novo-Helisen**	**Acarizax Orylmyte**

Bold = authorized preparations; *No authorized preparation. The AIT products are listed in alphabetical order and do not represent a ranking. The criterion for inclusion in the table is only marketing authorisation for the respective age group. Source: Paul Ehrlich Institut (accessed September 29, 2023).
